# Sarcopenia and diabetes-induced dementia risk

**DOI:** 10.1093/braincomms/fcad347

**Published:** 2023-12-20

**Authors:** Mingyang Sun, Zhongyuan Lu, Wan-Ming Chen, Szu-Yuan Wu, Jiaqiang Zhang

**Affiliations:** Department of Anesthesiology and Perioperative Medicine, People's Hospital of Zhengzhou University, Henan Provincial People's Hospital, Zhengzhou, Henan 450052 China; Academy of Medical Sciences of Zhengzhou University, Zhengzhou, Henan 450052, China; Department of Anesthesiology and Perioperative Medicine, People's Hospital of Zhengzhou University, Henan Provincial People's Hospital, Zhengzhou, Henan 450052 China; Academy of Medical Sciences of Zhengzhou University, Zhengzhou, Henan 450052, China; Graduate Institute of Business Administration, College of Management, Fu Jen Catholic University, Taipei 242, Taiwan; Artificial Intelligence Development Center, Fu Jen Catholic University, Taipei 242, Taiwan; Graduate Institute of Business Administration, College of Management, Fu Jen Catholic University, Taipei 242, Taiwan; Artificial Intelligence Development Center, Fu Jen Catholic University, Taipei 242, Taiwan; Department of Food Nutrition and Health Biotechnology, College of Medical and Health Science, Asia University, Taichung 413, Taiwan; Big Data Center, Lo-Hsu Medical Foundation, Lotung Poh-Ai Hospital, Yilan 265, Taiwan; Division of Radiation Oncology, Lo-Hsu Medical Foundation, Lotung Poh-Ai Hospital, Yilan 265, Taiwan; Department of Healthcare Administration, College of Medical and Health Science, Asia University, Taichung 413, Taiwan; Cancer Center, Lo-Hsu Medical Foundation, Lotung Poh-Ai Hospital, Yilan 265, Taiwan; Centers for Regional Anesthesia and Pain Medicine, Taipei Municipal Wan Fang Hospital, Taipei Medical University, Taipei 110, Taiwan; Department of Management, College of Management, Fo Guang University, Yilan 262, Taiwan; Department of Anesthesiology and Perioperative Medicine, People's Hospital of Zhengzhou University, Henan Provincial People's Hospital, Zhengzhou, Henan 450052 China; Academy of Medical Sciences of Zhengzhou University, Zhengzhou, Henan 450052, China

**Keywords:** old-age, T2DM, sarcopenia, diabetes-associated dementia, risk factors

## Abstract

This study aimed to investigate whether sarcopenia independently increases the risk of diabetes-induced dementia in elderly individuals diagnosed with type 2 diabetes mellitus. The study cohort consisted of a large sample of elderly individuals aged 60 years and above, who were diagnosed with type 2 diabetes mellitus between 2008 and 2018. To minimize potential bias and achieve covariate balance between the sarcopenia and non-sarcopenia groups, we employed propensity score matching. Various statistical analyses, including Cox regression models to assess dementia risk and associations, competing risk analysis to account for mortality and Poisson regression analysis for incidence rates, were used. Before propensity score matching, the study included 406 573 elderly type 2 diabetes mellitus patients, with 20 674 in the sarcopenia group. Following propensity score matching, the analysis included a total of 41 294 individuals, with 20 647 in the sarcopenia group and 20 647 in the non-sarcopenia group. Prior to propensity score matching, elderly type 2 diabetes mellitus patients with sarcopenia exhibited a significantly higher risk of dementia (adjusted hazard ratio: 1.12, 95% confidence interval: 1.07–1.17). After propensity score matching, the risk remained significant (adjusted hazard ratio: 1.14, 95% confidence interval: 1.07–1.21). Incidence rates of dementia were notably higher in the sarcopenia group both before and after propensity score matching, underscoring the importance of sarcopenia as an independent risk factor. Our study highlights sarcopenia as an independent risk factor for diabetes-induced dementia in elderly type 2 diabetes mellitus patients. Advanced age, female gender, lower income levels, rural residency, higher adapted diabetes complication severity index and Charlson Comorbidity Index scores and various comorbidities were associated with increased dementia risk. Notably, the use of statins was linked to a reduced risk of dementia. This research underscores the need to identify and address modifiable risk factors for dementia in elderly type 2 diabetes mellitus patients, offering valuable insights for targeted interventions and healthcare policies.

## Teaser

Sun *et al*. find sarcopenia independently raises dementia risk in elderly type 2 diabetes mellitus patients. Following propensity score matching, sarcopenic elderly type 2 diabetes mellitus patients had notably higher dementia risk, emphasizing the importance of addressing modifiable dementia risk factors in this population.

## Graphical Abstract

**Graphical Abstract:**
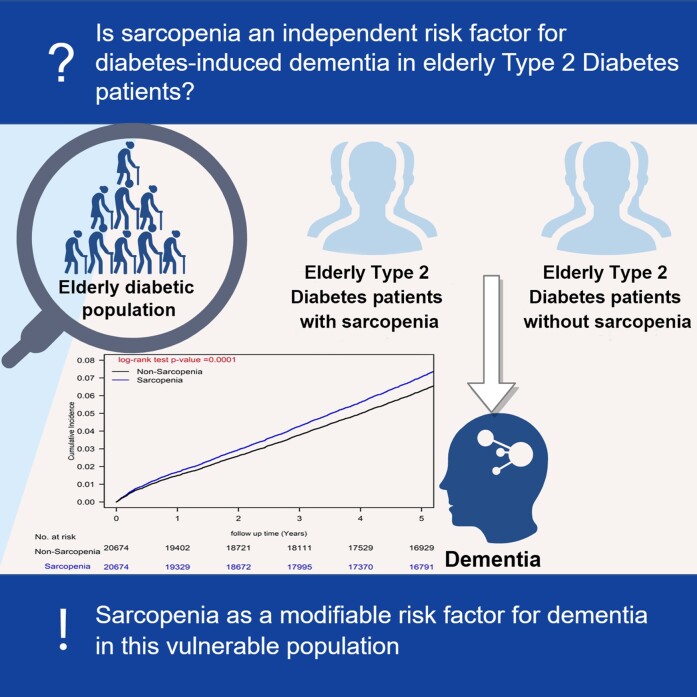

## Introduction

Diabetes mellitus is a pervasive and chronic metabolic disorder that afflicts millions of individuals worldwide,^1^ posing significant healthcare challenges and economic burdens.^2^ Among the manifold complications entwined with diabetes mellitus, dementia has arisen as an escalating concern, casting substantial societal and economic tolls. This issue is particularly pronounced among the elderly population afflicted with type 2 diabetes mellitus (T2DM). With the global demographic shift towards an aging society and the concurrent surge in T2DM prevalence, the nexus between diabetes and dementia becomes increasingly pertinent. The elderly population with T2DM is particularly susceptible to dementia, and with the global aging trend and increasing T2DM prevalence, this issue is of growing concern.^3,4^ Among the complications linked to T2DM, dementia stands out as a significant concern due to its impact on cognitive function and daily life. Dementia poses substantial economic burdens due to the need for long-term care, extensive medical services and its impact on social welfare systems.^5,6^ The economic burden of dementia is substantial, as it requires extensive medical care, long-term care and places a strain on social welfare systems.^5,6^

Sarcopenia, characterized by age-related loss of skeletal muscle mass and function, is prevalent among elderly individuals, including those with T2DM.^7,8^ Sarcopenia not only affects physical health and independence but has also been associated with cognitive decline and an elevated risk of dementia.^9-13^ The interconnectedness of sarcopenia, old age, T2DM and dementia highlights the importance of understanding their relationships and implications. Elderly individuals with T2DM are at a higher risk of experiencing both sarcopenia and dementia,^3,14^ potentially forming a pathophysiologic continuum. Sarcopenia, characterized by age-related muscle loss, plays a pivotal role in a pathophysiologic continuum connecting T2DM and dementia. It acts as a mediator between the two conditions, with reduced muscle mass and function contributing to insulin resistance and increased T2DM risk. Subsequently, T2DM exacerbates cognitive decline and dementia risk, illustrating the intricate interplay of these health issues within a broader continuum of pathophysiologic processes. Sarcopenia may act as a mediator between T2DM and dementia, exacerbating cognitive decline in this vulnerable population. Recent studies have increasingly focused on the potential connection between sarcopenia and dementia, making this a noteworthy area of investigation. Therefore, given the intricate relationship between sarcopenia, aging, T2DM and dementia, further investigation is warranted to better understand these connections and their implications for elderly T2DM patients. Understanding these connections can inform the development of comprehensive strategies for promoting healthy aging and preventing dementia in elderly individuals with T2DM. By addressing sarcopenia as a modifiable risk factor, healthcare professionals can play a pivotal role in enhancing the overall well-being and quality of life for this vulnerable population. If sarcopenia indeed proves to be a risk factor for diabetes-induced dementia, it would be of significant importance, as sarcopenia is a modifiable condition that can be prevented or delayed through lifestyle interventions.

Against this backdrop, our study endeavours to investigate the independent role of sarcopenia as a risk factor for diabetes-induced dementia in elderly T2DM patients. By delving into the potential association between sarcopenia and dementia, we aspire to enrich the understanding of dementia risk factors within this vulnerable population. This research is pivotal for guiding proactive measures and targeted interventions aimed at alleviating the burden of diabetes-induced dementia and fostering healthy aging among elderly individuals with T2DM. In this research, we hypothesize that sarcopenia is an independent risk factor for diabetes-induced dementia in elderly individuals diagnosed with T2DM. We also anticipate that elderly individuals with T2DM who exhibit sarcopenia will demonstrate a higher incidence of dementia compared with those without sarcopenia. These hypotheses provide a clear framework for our research and guide readers in understanding our objectives and expected outcomes. Through rigorous investigation and analysis, we aim to either support or refute these hypotheses, thereby advancing our understanding of the complex relationships between sarcopenia, T2DM and dementia in elderly populations.

## Patients and methods

### Study population

Conducted within a population-based cohort design, this study utilized the extensive National Health Insurance Research Database in Taiwan.^15-17^ This database contains comprehensive medical and demographic information while ensuring patient privacy. The study focused on individuals aged 60 or older diagnosed with T2DM between 2008 and 2018. Stringent data integrity measures were applied, including the exclusion of patients with incomplete age-related data or pre-existing dementia. The cohort was divided into sarcopenia and non-sarcopenia groups using propensity score matching (PSM), with the index date set as the sarcopenia diagnosis date. Dementia cases occurring before or within 1 year of the index date were excluded. Ethical considerations have been diligently addressed as well, with the study's protocols having obtained the requisite approval from the Institutional Review Board of Tzu-Chi Medical Foundation (IRB109-015-B), further reinforcing the scholarly rigour and ethical compliance of this investigation.

In the context of Taiwan, the conceptualization of sarcopenia aligns with our prior scholarly works,^18-22^ predominantly relying on the diagnostic codes furnished by family medicine practitioners, rehabilitation specialists, neurologists and neurosurgeons under the ICD-9 and ICD-10 classification systems. The classification of dementia cases underwent a granular approach, delineating them into distinct subgroups according to specific subtypes, including Alzheimer's disease, vascular dementia and other variants of dementia. To establish a definitive diagnosis of dementia, a minimum of three visits within a consecutive year documented in the National Health Insurance Research Database was mandated, coupled with the endorsement of specialists in psychiatry, neurology or psychosomatic medicine, thereby ensuring the robustness of the diagnostic criteria.

### Study covariates and PSM

In this comprehensive study, we have meticulously selected a range of covariates, each bearing significant relevance to our investigation into the independent risk of diabetes-induced dementia associated with sarcopenia in elderly individuals diagnosed with T2DM. The inclusion of these covariates is paramount, as they collectively illuminate the intricate web of factors contributing to both treatment assignment and dementia outcomes within this vulnerable population. Age, a cornerstone covariate, inherently embodies the increased susceptibility of the elderly to sarcopenia and dementia,^23,24^ forming the foundation for our research. Gender, income level and urbanization degree, in turn, delineate socio-economic disparities and lifestyle variances that can profoundly influence the prevalence and severity of both sarcopenia and dementia.^13,25-28^ The choice of antidiabetic medications and specific agents therein is not merely a surrogate for diabetes severity but also a dynamic determinant of treatment response, necessitating nuanced examination.^29^ The adapted diabetes complication severity index (aDCSI) score offers a quantitative lens through which to gauge the severity of diabetes-related complications,^30^ reflecting their direct impact on both sarcopenia and dementia.^28^ Concurrent comorbidities linked to dementia risk,^31-39^ smoking status,^40^ occurrences of alcohol-related liver conditions^41^ and Charlson Comorbidity Index (CCI) scores all embody distinct facets of health status and lifestyle choices that can sway the trajectory of these intertwined conditions.^42^ Lastly, the influence of various pharmaceuticals, such as statins, anticholinergic agents, benzodiazepines and antipsychotics, introduces multifaceted interactions that may either mitigate or exacerbate the risk of diabetes-induced dementia in elderly T2DM patients.^43-47^ By meticulously considering these covariates, our study aims to unravel the complex interplay between sarcopenia, T2DM and dementia, and offering valuable insights for targeted interventions and healthcare policies.

The elderly cohort was categorized into age brackets, and we used PSM to balance covariates between sarcopenia and non-sarcopenia diabetes groups. Variables included age, gender, income level, urbanization degree, antidiabetic medications, aDCSI, comorbidities, smoking, alcohol-related liver conditions, CCI scores and medications such as statins. We avoided redundancy in our analyses by carefully removing duplicated comorbidities when calculating CCI scores. Comorbidities were identified using diagnostic codes from inpatient records or if there were at least two outpatient visits within a year, with a focus on comorbidities occurring in the year leading up to the index date. We also presented continuous variables in a clear manner, using descriptions like mean ± standard deviation or median (first quartile and third quartile) as appropriate.

### Outcome variables

The central objective of this study was the assessment of dementia risk as the primary outcome variable. The manifestation of dementia was meticulously monitored from the index date, persisting until either the date of dementia diagnosis or the conclusion of the study period, spanning up to 30 December 2021. Additionally, we investigated secondary outcome measures, encompassing the scrutiny of the incidence rate (IR) and IR ratio (IRR) of dementia within our cohort. For the estimation of the IRR pertaining to dementia, we employed a rigorous statistical approach, namely, Poisson regression analysis.

### Statistical analysis

To comprehensively address potential confounding factors, we employed Cox regression models with meticulous adjustments involving a broad spectrum of covariates. These covariates encompassed key variables such as age, sex, income level, urbanization level, types of antidiabetic drugs used (as a surrogate for diabetes severity), specific antidiabetic drugs, aDCSI score, presence of coexisting comorbidities associated with dementia risk, smoking status, presence of alcohol-related liver diseases, CCI scores and other relevant medications.^48^ Furthermore, we adopted competing risk analysis to account for the risk of mortality, as indicated in Tables 1 and 2. The cumulative incidences of dementia were evaluated using the Kaplan–Meier method, and distinctions between the sarcopenia and non-sarcopenia groups were assessed through a stratified log-rank test, as presented in Supplemental Fig. 1. In order to ensure methodological rigour and statistical robustness, all analytical procedures were conducted using SAS software (version 9.4; SAS Institute, Cary, NC, USA).

**Table 1:** Dementia risk and adjusted hazard ratios in elderly patients with T2DM: a comparison between sarcopenia and non-sarcopenia groups before PSM

Variable	Crude HR (95% CI)		*P*	Adjusted HR (95% CI)^a^		*P*
**Sarcopenia status**						
Non-sarcopenia	**Reference**					
Sarcopenia	1.46	(1.4, 1.53)	<0.0001	1.12	(1.07, 1.17)	<0.0001
**Sarcopenia status** ^b^						
Non-sarcopenia	**Reference**					
Sarcopenia	1.46	(1.4, 1.53)	<0.0001	1.11	(1.07, 1.15)	<0.0001

CI, confidence interval; HR, hazard ratio. ^a^ Adjusted for all covariates shown in Supplemental Table 1 using a Cox proportional regression model. ^b^ Adjusted for all covariates shown in Supplemental Table 1 using a Cox proportional regression model with competing risk of mortality.

**Table 2:** Dementia risk and adjusted hazard ratios in elderly patients with T2DM: a comparison between sarcopenia and non-sarcopenia groups after PSM

Variable	Crude HR (95% CI)		*P*	Adjusted HR (95% CI)^a^		*P*
**Sarcopenia status**						
Non-sarcopenia	**Reference**					
Sarcopenia	1.13	(1.06, 1.2)	0.0001	1.14	(1.07, 1.21)	<0.0001
**Sarcopenia status** ^b^						
Non-sarcopenia	**Reference**					
Sarcopenia	1.13	(1.06, 1.2)	0.0001	1.13	(1.06, 1.21)	0.0001

CI, confidence interval; HR, hazard ratio. ^a^ Adjusted for all covariates shown in Supplemental Table 1 using a Cox proportional regression model. ^b^ Adjusted for all covariates shown in Supplemental Table 1 using a Cox proportional regression model with competing risk of mortality.

### Ethics approval and consent

The study protocols were reviewed and approved by the Institutional Review Board of Tzu-Chi Medical Foundation (IRB109-015-B).

## Results

Prior to PSM, the study included a total of 406 573 elderly patients diagnosed with T2DM, among whom 385 899 were classified into the non-sarcopenia group, while the remaining 20 674 were categorized as having sarcopenia. Within the sarcopenia group, a higher prevalence of elderly individuals was observed, along with a greater proportion of females, lower income levels, rural residency, a decreased utilization of various types of antidiabetic drugs and reduced use of insulin, metformin, sulfonylureas and alpha-glucosidase inhibitors. Moreover, individuals within the sarcopenia group exhibited elevated aDCSI scores and a heightened prevalence of coexisting comorbidities. Furthermore, the sarcopenia group displayed a higher proportion of cigarette smokers, cases of alcohol-related liver diseases and utilization of medications such as statins, anticholinergic drugs, benzodiazepines and antipsychotics, compared with their non-sarcopenia T2DM counterparts (as detailed in Supplemental Table 1).

Subsequent to PSM, our investigation encompassed a total of 41 294 elderly individuals residing in Taiwan, all of whom received a T2DM diagnosis between 2008 and 2018. The mean age at the time of T2DM diagnosis was 86.31 years for the non-sarcopenia group and 86.97 years for the sarcopenia group. Through scrupulous analysis and refinement, we successfully achieved a well-distributed alignment of all covariates between the two aforementioned groups. This outcome attests to the efficacy of our meticulous adjustment procedure in attaining a state of covariate equivalence, as delineated in Supplemental Table 1. Importantly, all calculated *P*-values surpassed the threshold of 0.05, thereby confirming the comparability between the two groups.

### Dementia risk and hazard ratios in sarcopenia and non-sarcopenia elderly patients with T2DM

Before implementing PSM, we noted a significant elevation in the risk of dementia among elderly individuals affected by sarcopenia, as indicated by an adjusted hazard ratio (aHR) of 1.12 [95% confidence interval (CI): 1.07–1.17; Table 1]. Subsequent to meticulous adjustment for all covariates, a process outlined in Supplemental Table 1, utilizing a Cox proportional regression model incorporating the competing risk of mortality (Table 1), our analysis showcased a substantial aHR of 1.11 (95% CI: 1.07–1.15) associated with dementia in the context of elderly individuals with both T2DM and sarcopenia.

Even following the implementation of PSM, our investigation sustained the observation of a significant elevation in the risk of dementia among elderly T2DM individuals with sarcopenia, as reflected in the aHR of 1.14 (95% CI: 1.07–1.21; Table 2). This relationship was further fortified by a notable log-rank test outcome (*P* = 0.0001; Supplemental Fig. 1). Moreover, even after rigorous covariate adjustment (as elucidated in Supplemental Table 1) using a Cox proportional regression model inclusive of the competing risk of mortality (Table 2), our analysis disclosed a noteworthy aHR of 1.13 (95% CI: 1.06–1.21) for dementia among elderly individuals with both T2DM and sarcopenia.

### Dementia incidence in elderly T2DM patients with sarcopenia

Within our elderly cohort afflicted by T2DM, a marked discrepancy in the incidence of dementia was observed between individuals subjected to PSM with non-sarcopenia (7.16%; 1478 individuals) and those with sarcopenia (10.00%; 2067 individuals), signifying a substantial divergence (*P* < 0.0001; Supplemental Table 1). Notably, a comparison of the IRs of dementia between sarcopenia and non-sarcopenia individuals, as demonstrated in Table 3, revealed higher IRs associated with sarcopenia. Prior to PSM, the analysis of IRRs further underscored this association, illustrating a calculated IRR (95% CI) of 1.45 (1.39–1.52) for sarcopenia in relation to non-sarcopenia. After PSM, the IRR analysis reiterated this relationship, indicating an IRR (95% CI) of 1.16 (1.02–1.20) for sarcopenia relative to non-sarcopenia. Specifically, before PSM, the IR for dementia in the non-sarcopenia group was 95.48 per 10 000 person-years, while the sarcopenia group exhibited an IR of 138.61 per 10 000 person-years. Following PSM, the IR for dementia in the non-sarcopenia group was 113.22 per 10 000 person-years, while the sarcopenia group retained an IR of 138.61 per 10 000 person-years.

**Table 3:** IRs and IRRs of dementia in elderly patients with T2DM: a comparison between sarcopenia and non-sarcopenia groups

Variables	Events	Person-years	IR (10 000 person-years)	IRR	95% CI for IRR	*P*
**Before propensity score matching**						
Non-sarcopenia	28 076	2 940 408	95.48	**Reference**		
Sarcopenia	2067	149 122	138.61	1.45	(1.39, 1.52)	<0.0001
**After propensity score matching**						
Non-sarcopenia	1478	153 709	113.22	**Reference**		
Sarcopenia	2067	149 122	138.61	1.12	(1.06, 1.20)	0.0002

CI, confidence interval; IR, incidence rate; IRR, incidence rate ratio.

### Hazard ratios of dementia risk in elderly T2DM patients before PSM

To comprehensively assess the risk factors contributing to dementia among elderly individuals with T2DM, we deployed a multivariate Cox proportional model to derive aHRs for dementia within the cohort prior to PSM. Following PSM, no statistically significant discrepancies were identified in the confounding factors, validating the appropriateness of employing the pre-PSM cohort for analysis. Our investigation into the risk factors associated with dementia in elderly T2DM patients involved the utilization of a multivariate Cox proportional model to compute aHRs. Noteworthy risk factors linked to an elevated dementia risk encompassed advanced age (with aHRs of 1.72, 3.86 and 12.37 for age groups 66–70, 71–75 and >75 years, respectively), female gender, lower income levels, rural residency, heightened aDCSI and CCI scores, along with comorbidities such as hypertension, hyperlipidaemia, stroke, depression, anxiety, peripheral vascular disease, Chronic obstructive pulmonary disease, traumatic head injury, hearing loss, liver cirrhosis and Systemic Lupus Erythematosus. Moreover, cigarette smoking and alcohol-related liver diseases emerged as contributors to increased dementia risk. Notably, the utilization of statins among elderly T2DM patients was associated with a significantly diminished risk of dementia in comparison with non-statin users. Please refer to Supplemental Table 2 for a comprehensive breakdown of the results.

## Discussion

In our population-based cohort study, we investigated the association between sarcopenia and dementia risk in elderly individuals with T2DM. To our knowledge, this is one of the first studies to focus specifically on the elderly T2DM population and examine the role of sarcopenia as a risk factor for dementia. Our findings revealed that elderly T2DM patients with sarcopenia had a significantly higher risk of developing dementia compared with those without sarcopenia. This novel finding highlights the potential importance of considering sarcopenia as a modifiable risk factor for dementia in this vulnerable population. After PSM, we found a significant increase in dementia risk among elderly T2DM individuals with sarcopenia, with an aHR of 1.14 (95% CI: 1.07–1.21; Table 2). This association was further supported by a remarkable log-rank test result (*P* = 0.0001; Supplemental Fig. 1). Even after meticulous adjustment for all covariates (as shown in Supplemental Table 1) using a Cox proportional regression model with competing risk of mortality (Table 2), our analysis unveiled a noteworthy aHR of 1.13 (95% CI: 1.06–1.21) for dementia among elderly T2DM individuals with sarcopenia, highlighting the novel link between sarcopenia and an increased risk of dementia in this specific population.

The relationship between sarcopenia and dementia in elderly T2DM patients is complex and likely influenced by various factors. Sarcopenia involves a gradual loss of muscle mass and function, leading to reduced physical activity and more time spent sitting.^7,49^ The reduced muscle strength and physical inactivity have been associated with impaired cerebral blood flow, altered neurotrophic factors and increased brain atrophy, all of which contribute to the development of cognitive decline and dementia.^50^ Moreover, both sarcopenia and T2DM share common features of chronic inflammation and insulin resistance, which may lead to neuroinflammation and neurodegeneration, further heightening the risk of dementia.^51,52^ In addition, insulin-like growth factor 1 and its signalling pathways play essential roles in maintaining the health of both muscles and the brain. Insulin-like growth factor 1 may serve as a potential link between sarcopenia, diabetes and cognitive decline.^53^ In short, sarcopenia and T2DM share pathways like insulin-like growth factor 1 and insulin resistance. Sarcopenia also increases dementia risk by reducing physical activity, which impacts cerebral blood flow. As a result, our study finds that sarcopenia is an independent risk factor for diabetes-induced dementia.

Preventing sarcopenia in elderly individuals with T2DM is of paramount importance, given the significant impact it can have on reducing the risk of diabetes-induced dementia, which imposes considerable economic, social and familial burdens as evidenced by the current study's findings (Tables 1 and 2). Dementia is associated with escalated healthcare costs, strains on social care systems and emotional and financial challenges for caregiving families.^54^ To effectively address this issue, the implementation of health policies and interventions aimed at preventing sarcopenia in T2DM patients becomes crucial. Promoting physical activity, including both aerobic exercise and resistance training, plays a pivotal role in maintaining muscle mass and function in elderly T2DM patients.^55^ Ensuring proper nutrition with adequate protein intake can significantly support muscle health, and optimizing glycaemic control can effectively reduce insulin resistance, thereby preventing the development of sarcopenia in this vulnerable population.^8,56^ Furthermore, the implementation of fall prevention strategies, providing cognitive stimulation, conducting regular medication reviews and offering multidisciplinary care can collectively contribute to preventing sarcopenia and reducing the risk of dementia among elderly T2DM patients.^57^ To achieve these preventive goals, public awareness and educational campaigns are essential to empower individuals to take proactive measures against diabetes-induced dementia.^5^ Preventive measures for dementia in elderly T2DM patients should focus on addressing modifiable risk factors. These measures include promoting physical activity and managing glycaemic control to reduce the risk of both T2DM and sarcopenia. Additionally, regular cognitive assessments and early interventions for cognitive decline should be considered. Healthcare policies should prioritize comprehensive care for elderly T2DM patients, including strategies to prevent and manage sarcopenia and dementia. Preventing sarcopenia and reducing diabetes-induced dementia in elderly T2DM patients involves promoting physical activity, ensuring adequate nutrition, optimizing glycaemic control, fall prevention, cognitive stimulation, medication reviews and multidisciplinary care. Public awareness campaigns are essential to encourage proactive measures.

Our results are consistent with some previous studies that have reported an association between sarcopenia and cognitive decline or dementia in older adults without diabetes.^9-13^ However, our study specifically focused on elderly individuals with T2DM, a population known to have an increased risk of cognitive impairment and dementia.^58^ Despite the unique characteristics of our study population, our findings align with the growing body of evidence suggesting that sarcopenia may be a common risk factor for cognitive decline, irrespective of diabetes status.^9-13^ In Supplemental Table 2, our findings were consistent with previous studies regarding several risk factors associated with dementia in elderly T2DM patients. Advanced age showed a dose-dependent relationship with dementia risk, with significantly elevated aHRs of 1.72, 3.86 and 12.37 for age groups 66–70, 71–75 and >75 years, respectively.^59^ Female gender,^60^ lower income levels^61^ and rural residence^62^ were also identified as consistent risk factors for dementia in this population. Our study also revealed novel findings regarding other risk factors not extensively explored in previous research. Higher aDCSI and CCI scores were associated with increased dementia risk, suggesting that the severity of diabetic complications and overall comorbidities could play a role in dementia development in elderly T2DM patients. In this specific population of elderly individuals with T2DM, several comorbidities, including hypertension, hyperlipidaemia, stroke, depression, anxiety, peripheral vascular disease, COPD, traumatic head injury, hearing loss, liver cirrhosis and SLE, were identified as significant risk factors for dementia. These factors, as shown in Supplemental Table 2, significantly contribute to the heightened risk of developing diabetes-induced dementia in old-age T2DM patients. Interestingly, prior research has relatively neglected these factors as exacerbators of dementia risk in elderly individuals with T2DM. Furthermore, an elevated risk of dementia was observed in connection with cigarette smoking and alcohol-related liver diseases.^40,63^ This underscores the significance of potential modifiable risk factors that could be targeted in dementia prevention strategies, such as smoking cessation and alcohol moderation. One notable difference in our study compared with others is the significant association between statin use and a reduced risk of dementia in elderly T2DM patients. This finding suggests a potential protective effect of statins against dementia development, which merits further investigation. While previous studies have explored the link between statins and cognitive function,^64,65^ our study specifically focused on the elderly T2DM population and demonstrated a noteworthy reduction in dementia risk among statin users. Our study reinforces the importance of age, gender, income levels, rural residence, diabetic complication severity and comorbidities as risk factors for dementia in elderly T2DM patients, consistent with existing literature.^40,59-67^ Additionally, we identify novel associations between cigarette smoking, alcohol-related liver diseases and statin use with dementia risk in this population. These findings contribute to a deeper understanding of the risk factors for diabetes-induced dementia and can inform targeted interventions to prevent sarcopenia in T2DM patients, especially in old age.

Our study possesses several notable strengths that contribute to its significance and reliability. First, this is the first article to demonstrate that sarcopenia is an independent risk factor for diabetes-induced dementia. Second, the utilization of a large sample size and the comprehensive and accurate National Health Insurance Research Database of Taiwan provided us access to a diverse and representative population, enhancing the generalizability of our findings to elderly T2DM patients. Third, the meticulous use of PSM allowed us to create a well-matched control group, effectively addressing potential confounding factors and ensuring the validity of our results. Fourth, our study's contribution to the existing literature is strengthened by the identification of new risk factors associated with diabetes-induced dementia in elderly T2DM patients. Through a multivariate Cox proportional model, we explored a wide range of potential risk factors, uncovering novel associations that have not been extensively investigated in previous studies. The inclusion of female, higher aDCSI and CCI scores, hypertension, hyperlipidaemia, stroke, depression, anxiety, peripheral vascular disease, COPD, traumatic head injury, hearing loss, liver cirrhosis and SLE as risk factors for dementia in this population expands our understanding of the intricate interplay between diabetes and cognitive impairment.^27-42^ Fifth, the discovery of statin use as a potential protective factor against dementia in elderly T2DM patients adds valuable insights and warrants further investigation into the neuroprotective effects of statins in this specific population. Overall, the comprehensive evaluation of multiple risk factors and the novel findings enhance the significance of our study, providing valuable guidance for preventive strategies to alleviate the burden of diabetes-induced dementia, particularly through the management of sarcopenia in elderly T2DM patients.

Despite its strengths, our study also has limitations. First, the retrospective nature of the study design may introduce inherent biases, and causality cannot be established. Second, it is important to acknowledge that the utilization of administrative databases could potentially lead to instances of underreporting or misclassification of specific variables, such as the diagnoses of sarcopenia or dementia. However, it is worth noting that if the diagnosis of sarcopenia is less frequent (but cases diagnosed as sarcopenia are certainly indicative of the condition), this implies that individuals with non-sarcopenic conditions might be included among those with sarcopenia, ultimately resulting in an underestimation of the dementia risk associated with sarcopenia. Consequently, this would suggest that the overall conclusion remains unchanged, adhering to the null hypothesis. Third, some potential confounders, such as physical activity level or dietary habits, were not available in the database, limiting our ability to account for their influence on the outcomes. Additionally, this study primarily focused on an Asian population, and extrapolating the results to other ethnic groups, particularly individuals of European or American descent, may require further validation. Lastly, while PSM reduced selection bias, residual confounding remains possible, and other unmeasured factors may still influence the observed associations.^68^ Future prospective studies with a more comprehensive assessment of risk factors and a diverse population are needed to confirm and extend our findings. Despite these limitations, our large study contributes valuable insights into the relationship between sarcopenia and diabetes-induced dementia in elderly T2DM patients, paving the way for further research in this critical area.

## Conclusions

Our study demonstrates that sarcopenia is an independent risk factor for diabetes-induced dementia in elderly patients with T2DM. We identified new risk factors associated with dementia in this population, including female, higher aDCSI and CCI scores, hypertension, hyperlipidaemia, stroke, depression, anxiety, peripheral vascular disease, COPD, traumatic head injury, hearing loss, liver cirrhosis and SLE. Preventive strategies targeting sarcopenia and other risk factors could play a pivotal role in reducing the burden of diabetes-induced dementia in elderly T2DM patients.

## Supplementary Material

fcad347_Supplementary_Data

## Data Availability

The data supporting the study's conclusions are included in the manuscript and were obtained from the National Health Insurance Research Database and Taiwan Cancer Registry database. However, due to the Personal Information Protection Act enforced by the Taiwanese government since 2012, there are restrictions on data access. The data used in this study cannot be publicly shared in the manuscript, supplemental files or any public repository. Interested parties can submit a formal proposal to request data access, which requires approval from the ethics review committee of the relevant governmental department in Taiwan. For more information on how to request data access, please visit the following links: http://nhird.nhri.org.tw/en/Data_Subsets.html#S3 and http://nhis.nhri.org.tw/point.html. Informed consent was waived because the data are covered by the Personal Information Protection Act. S.-Y.W., MD, PhD, had complete access to all the study data and assumes responsibility for data integrity and accuracy. The data have not been previously presented at scientific meetings in oral or poster form.
