# Supplementary material for: Sarcopenia and diabetes-induced dementia risk
Source: Brain Commun. 2023 Dec 20;6(1):fcad347. doi: 10.1093/braincomms/fcad347 (PMC10766377; doi:10.1093/braincomms/fcad347)
Supplement: fcad347_Supplementary_Data [file fcad347_supplementary_data.docx]

**Supplemental** **Table 1.** Baseline Characteristics of Elderly Type 2 Diabetes Patients with and without Sarcopenia

|  | **Before Propensity Scores Matching** | | | | | **After Propensity Scores Matching** | | | |  |
| --- | --- | --- | --- | --- | --- | --- | --- | --- | --- | --- |
|  | **Non-Sarcopenia** | | **Sarcopenia** | | ***P*** | **Non-Sarcopenia** | | **Sarcopenia** | | ***P*** |
|  | **N=385,899** | | **N=20,674** | |  | **N=20,674** | | **N=20,674** | |  |
|  | **N** | **%** | **N** | **%** |  | **N** | **%** | **N** | **%** |  |
| **Age** (mean ± SD) | 77.36 ± 13.50 | | 86.79 ± 13.79 | | <0.0001 | 86.31 ± 13.24 | | 86.97 ± 13.29 | | 0.2122 |
| Age, median (IQR), years-old | 78.00 (73.00,86.00) | | 86.00 (79.00,91.00) | | <0.0001 | 86.00 (77.00,95.00) | | 86.00 (78.00,96.00) | | 0.3215 |
| Age group, years-old |  |  |  |  | <0.0001 | 0.8558 |  |  |  | 0.0980 |
| 60-65 | 88,222 | 22.86% | 3,343 | 16.17% |  | 3,290 | 15.91% | 3,343 | 16.17% |  |
| 66-70 | 106,950 | 27.71% | 5,412 | 26.18% |  | 5,403 | 26.13% | 5,412 | 26.18% |  |
| 71-75 | 86,048 | 22.30% | 5,132 | 24.82% |  | 5,126 | 24.79% | 5,132 | 24.82% |  |
| >75 | 104,679 | 27.13% | 6,787 | 32.83% |  | 6,855 | 33.16% | 6,787 | 32.83% |  |
| **Sex** |  |  |  |  | <0.0001 |  |  |  |  | 0.7971 |
| Female | 182,310 | 47.24% | 11,666 | 56.43% |  | 11,637 | 56.29% | 11,666 | 56.43% |  |
| Male | 203,589 | 52.76% | 9,008 | 43.57% |  | 9,037 | 43.71% | 9,008 | 43.57% |  |
| **Income level (NTD)** |  |  |  |  | <0.0001 |  |  |  |  | 0.1631 |
| Low income | 5,675 | 1.47% | 322 | 1.56% |  | 276 | 1.34% | 322 | 1.56% |  |
| Financially dependent | 120,198 | 31.15% | 6,544 | 31.65% |  | 6,656 | 32.20% | 6,544 | 31.65% |  |
| ≤20 000 | 181,685 | 47.08% | 10,337 | 50.00% |  | 10,377 | 50.19% | 10,337 | 50.00% |  |
| 20 001-30 000 | 36,373 | 9.43% | 1,759 | 8.51% |  | 1,736 | 8.40% | 1,759 | 8.51% |  |
| 30 001-45 000 | 26,547 | 6.88% | 1,147 | 5.55% |  | 1,119 | 5.41% | 1,147 | 5.55% |  |
| >45 000 | 15,421 | 4.00% | 565 | 2.73% |  | 510 | 2.47% | 565 | 2.73% |  |
| **Urbanization level** |  |  |  |  | <0.0001 |  |  |  |  | 0.4314 |
| Rural | 107,253 | 27.79% | 7,500 | 36.28% |  | 7,577 | 36.65% | 7,500 | 36.28% |  |
| Urban | 278,646 | 72.21% | 13,174 | 63.72% |  | 13,097 | 63.35% | 13,174 | 63.72% |  |
| **Types of antidiabetic drugs used (Diabetes severity)** |  |  |  |  | <0.0001 |  |  |  |  | 0.0659 |
| 0 | 140,900 | 36.51% | 8,548 | 41.35% |  | 8,731 | 42.23% | 8,548 | 41.35% |  |
| 1 | 96,159 | 24.92% | 5,117 | 24.75% |  | 5,046 | 24.41% | 5,117 | 24.75% |  |
| 2 | 95,209 | 24.67% | 4,446 | 21.51% |  | 4,472 | 21.63% | 4,446 | 21.51% |  |
| 3 | 38,922 | 10.09% | 1,926 | 9.32% |  | 1,876 | 9.07% | 1,926 | 9.32% |  |
| ≥4 | 14,709 | 3.81% | 637 | 3.08% |  | 549 | 2.66% | 637 | 3.08% |  |
| **Antidiabetic drug** |  |  |  |  |  |  |  |  |  |  |
| Insulin | 52,058 | 13.49% | 2,659 | 12.86% | 0.0099 | 2,586 | 12.52% | 2,659 | 12.86% | 0.6100 |
| Metformin | 163,314 | 42.32% | 7,981 | 38.60% | <0.0001 | 7,880 | 38.17% | 7,981 | 38.60% | 0.3418 |
| Sulfonylureas | 184,356 | 47.77% | 8,577 | 41.49% | <0.0001 | 8,689 | 42.03% | 8,577 | 41.49% | 0.2640 |
| SGLT2 inhibitors | 2,909 | 0.75% | 191 | 0.92% | 0.0062 | 181 | 0.88% | 191 | 0.92% | 0.4059 |
| Alpha-glucosidase inhibitors | 23,550 | 6.10% | 1,156 | 5.59% | 0.0027 | 1,099 | 5.32% | 1,156 | 5.59% | 0.2170 |
| Thiazolidinediones | 17,249 | 4.47% | 731 | 3.54% | <0.0001 | 702 | 3.40% | 731 | 3.54% | 0.4356 |
| GLP-1 agonists | 257 | 0.07% | 28 | 0.14% | 0.0003 | 28 | 0.14% | 28 | 0.14% | 0.9999 |
| DPP4 inhibitors | 22,075 | 5.72% | 1,175 | 5.68% | 0.8236 | 1,168 | 5.66% | 1,175 | 5.68% | 0.8013 |
| **Diabetes severity** |  |  |  |  |  |  |  |  |  |  |
| **aDCSI Score** (mean ± SD) | 0.93 ± 1.30 | | 1.15 ± 1.40 | | <0.0001 | 1.14 ± 1.36 | | 1.15 ± 1.40 | | 0.4118 |
| Median (IQR, Q1-Q3) | 0.00 (0.00,2.00) | | 1.00 (0.00,2.00) | | <0.0001 | 1.00 (0.00,2.00) | | 1.00 (0.00,2.00) | | 0.4621 |
| aDCSI Score |  |  |  |  | <0.0001 |  |  |  |  | 0.1889 |
| 0 | 205,490 | 53.25% | 9,117 | 44.10% |  | 9,206 | 44.53% | 9,117 | 44.10% |  |
| 1 | 79,801 | 20.68% | 4,950 | 23.94% |  | 5,081 | 24.58% | 4,950 | 23.94% |  |
| 2 | 57,031 | 14.78% | 3,469 | 16.78% |  | 3,401 | 16.45% | 3,469 | 16.78% |  |
| 3 | 21,234 | 5.50% | 1,580 | 7.64% |  | 1,550 | 7.50% | 1,580 | 7.64% |  |
| ≥4 | 22,343 | 5.79% | 1,558 | 7.54% |  | 1,436 | 6.95% | 1,558 | 7.54% |  |
| aDCSI |  |  |  |  |  |  |  |  |  |  |
| Retinopathy | 20,113 | 5.21% | 1,004 | 4.86% | 0.0248 | 1,060 | 5.13% | 1,004 | 4.86% | 0.2060 |
| Nephropathy | 45,424 | 11.77% | 2,945 | 14.24% | <0.0001 | 2,897 | 14.01% | 2,945 | 14.24% | 0.4980 |
| Neuropathy | 36,596 | 9.48% | 2,792 | 13.50% | <0.0001 | 2,759 | 13.36% | 2,792 | 13.50% | 0.7762 |
| Cerebrovascular | 37,053 | 9.60% | 2,533 | 12.25% | <0.0001 | 2,458 | 11.89% | 2,533 | 12.25% | 0.2576 |
| Cardiovascular | 96,293 | 24.95% | 6,809 | 32.94% | <0.0001 | 6,830 | 33.04% | 6,809 | 32.94% | 0.8261 |
| Peripheral vascular disease | 14,534 | 3.77% | 884 | 4.28% | 0.0002 | 852 | 4.12% | 884 | 4.28% | 0.4326 |
| Metabolic | 8,367 | 2.17% | 375 | 1.81% | 0.0006 | 381 | 1.84% | 375 | 1.81% | 0.8257 |
| **Coexisting comorbidities** |  |  |  |  |  |  |  |  |  |  |
| Hypertension | 190,683 | 49.41% | 12,115 | 58.60% | <0.0001 | 12,170 | 58.87% | 12,115 | 58.60% | 0.5827 |
| Hyperlipidemia | 137,518 | 35.64% | 8,861 | 42.86% | <0.0001 | 8,880 | 42.95% | 8,861 | 42.86% | 0.8503 |
| Coronary artery disease | 81,283 | 21.06% | 6,391 | 30.91% | <0.0001 | 6,282 | 30.39% | 6,391 | 30.91% | 0.2450 |
| Stroke | 48,303 | 12.52% | 3,791 | 18.34% | <0.0001 | 3,787 | 18.34% | 3,791 | 18.34% | 0.8988 |
| Depression | 21,406 | 5.55% | 2,295 | 11.10% | <0.0001 | 2,180 | 10.54% | 2,295 | 11.10% | 0.0687 |
| Anxiety | 48,596 | 12.59% | 4,818 | 23.30% | <0.0001 | 4,806 | 23.25% | 4,818 | 23.30% | 0.8889 |
| Heart failure | 23,112 | 5.99% | 1,835 | 8.88% | <0.0001 | 1,805 | 8.74% | 1,835 | 8.88% | 0.3223 |
| Peripheral vascular disease | 7,392 | 1.92% | 691 | 3.34% | <0.0001 | 640 | 3.10% | 691 | 3.34% | 0.1553 |
| COPD | 73,349 | 19.01% | 6,123 | 29.62% | <0.0001 | 6,153 | 29.76% | 6,123 | 29.62% | 0.7468 |
| Atrial fibrillation | 7,444 | 1.93% | 557 | 2.69% | <0.0001 | 550 | 2.66% | 557 | 2.69% | 0.6826 |
| Traumatic head injury | 21,352 | 5.53% | 1,695 | 8.20% | <0.0001 | 1,667 | 8.07% | 1,695 | 8.20% | 0.4311 |
| Hearing loss | 9,302 | 2.41% | 835 | 4.04% | <0.0001 | 826 | 4.00% | 835 | 4.04% | 0.5949 |
| Sleep apnea | 2,010 | 0.52% | 165 | 0.80% | <0.0001 | 142 | 0.69% | 165 | 0.80% | 0.1876 |
| Liver Cirrhosis | 105,335 | 27.30% | 7,324 | 35.43% | <0.0001 | 7,417 | 35.88% | 7,324 | 35.43% | 0.3396 |
| Systemic Lupus Erythematosus | 5,543 | 1.44% | 584 | 2.82% | <0.0001 | 536 | 2.59% | 584 | 2.82% | 0.1459 |
| **Cigarette smoking** | 40,712 | 10.55% | 3,256 | 15.77% | <0.0001 | 3,229 | 15.64% | 3,256 | 15.77% | 0.6413 |
| **Alcohol liver diseases** | 31,296 | 8.11% | 2,387 | 11.56% | <0.0001 | 2,355 | 11.41% | 2,387 | 11.56% | 0.2853 |
| **Other Medications** |  |  |  |  |  |  |  |  |  |  |
| Statins | 98,316 | 25.48% | 5,832 | 28.21% | <0.0001 | 5,754 | 27.83% | 5,832 | 28.21% | 0.3930 |
| Anticholinergic drugs | 99,137 | 25.69% | 6,586 | 31.90% | <0.0001 | 6,478 | 31.34% | 6,586 | 31.90% | 0.5421 |
| Benzodiazepines | 30,833 | 7.99% | 2,668 | 12.92% | <0.0001 | 2,655 | 12.86% | 2,668 | 12.92% | 0.5033 |
| Antipsychotics | 42,333 | 10.97% | 2,496 | 12.09% | <0.0001 | 2,405 | 11.65% | 2,496 | 12.09% | 0.1126 |
| **CCI Scores** |  |  |  |  |  |  |  |  |  |  |
| Mean (SD) | 1.50 ± 1.69 | | 1.88 ± 1.88 | | <0.0001 | 1.87 ± 1.78 | | 1.88 ± 1.88 | | 0.5312 |
| Median (Q1-Q3) | 1.00 (0.00,2.00) | | 2.00 (0.00,3.00) | | <0.0001 | 2.00 (0.00,3.00) | | 2.00 (0.00,3.00) | | 0.5190 |
| CCI Scores |  |  |  |  | <0.0001 |  |  |  |  | 0.1717 |
| 0 | 153,441 | 39.76% | 6,379 | 30.86% |  | 6,251 | 30.24% | 6,379 | 30.86% |  |
| ≥1 | 232,458 | 60.24% | 14,295 | 69.14% |  | 14,423 | 69.76% | 14,295 | 69.14% |  |
| **Follow-up time** |  | | | | | | | | | |
| Mean (SD) follow-up year | 8.06 ± 2.29 | | 8.04 ± 3.11 | | 0.7860 | 8.01 ± 2.50 | | 8.04 ± 2.82 | | 0.7641 |
| Median (IQR) follow-up year | 8.36 (7.36,9.62) | | 8.26 (5.51,9.36) | | 0.7652 | 8.21 (7.24,9.56) | | 8.24 (7.03,9.54) | | 0.8862 |
| **Dementia** | 28,076 | 7.28% | 2,067 | 10.00% | <0.0001 | 1,478 | 7.16% | 2,067 | 10.00% | <0.0001 |

**Abbreviations:** SD, standard deviation; IQR, interquartile range; T2DM, type 2 diabetes mellitus; CCI, Charlson comorbidity index; NTD, New Taiwan dollar; aDCSI, adapted diabetic complication severity index; N, Number; SGLT2 inhibitors, Sodium-Glucose Co-Transporter 2 inhibitors; GLP-1 agonists, Glucagon-Like Peptide 1 agonists; DPP4 inhibitors, Dipeptidyl Peptidase-4 inhibitors; COPD, Chronic Obstructive Pulmonary Disease.

**Supplemental Table 2.** Cox Proportional Regression Model for Adjusted Hazard Ratios of Dementia Risk in Elderly Type 2 Diabetes Mellitus Patients before Propensity Score Matching

|  | **Crude HR( 95%CI )** | | **P-value** | **Adjusted HR^*^( 95%CI )** | | **P-value** |
| --- | --- | --- | --- | --- | --- | --- |
|  |  |  |  |  |  |  |
| **Sarcopenia** (ref. no) |  |  |  |  |  |  |
| Yes | 1.46 | (1.4, 1.53) | <0.0001 | 1.12 | (1.07, 1.17) | <0.0001 |
| **Age** group, years (ref. 60-65) |  |  |  |  |  |  |
| 66-70 | 1.89 | (1.77, 2.03) | <0.0001 | 1.72 | (1.61, 1.85) | <0.0001 |
| 71-75 | 5.06 | (4.75, 5.39) | <0.0001 | 3.86 | (3.62, 4.12) | <0.0001 |
| >75 | 20.67 | (19.49, 21.93) | <0.0001 | 12.37 | (11.61, 13.18) | <0.0001 |
| **Sex** (ref. Female ) |  |  |  |  |  |  |
| Male | 0.85 | (0.83, 0.87) | <0.0001 | 0.95 | (0.93, 0.97) | <0.0001 |
| **Income** **levels** (ref. low income), NTD |  |  |  |  |  |  |
| Financially dependent | 0.53 | (0.5, 0.57) | <0.0001 | 0.45 | (0.42, 0.48) | <0.0001 |
| ≤20 000 | 0.44 | (0.41, 0.47) | <0.0001 | 0.43 | (0.4, 0.45) | <0.0001 |
| 20 001-30 000 | 0.12 | (0.1, 0.13) | <0.0001 | 0.32 | (0.29, 0.35) | <0.0001 |
| 30 001-45 000 | 0.08 | (0.07, 0.08) | <0.0001 | 0.25 | (0.22, 0.28) | <0.0001 |
| >45 000 | 0.07 | (0.06, 0.08) | <0.0001 | 0.23 | (0.2, 0.27) | <0.0001 |
| **Urbanization** (ref. rural) |  |  |  |  |  |  |
| Urban | 0.71 | (0.69, 0.72) | <0.0001 | 0.97 | (0.94, 0.99) | 0.0050 |
| **aDCSI** (ref. aDCSI=0) |  |  |  |  |  |  |
| 1 | 1.66 | (1.61, 1.72) | <0.0001 | 1.11 | (1.07, 1.15) | <0.0001 |
| 2 | 2.98 | (2.89, 3.07) | <0.0001 | 1.43 | (1.38, 1.48) | <0.0001 |
| 3 | 4.17 | (4.01, 4.34) | <0.0001 | 1.57 | (1.5, 1.63) | <0.0001 |
| ≥4 | 6.98 | (6.73, 7.24) | <0.0001 | 2.04 | (1.95, 2.13) | <0.0001 |
| **Types of antidiabetic drugs used** (ref =0) |  |  |  |  |  |  |
| 1 | 0.97 | (0.94, 0.99) | 0.0183 | 0.94 | (0.83, 1.06) | 0.3011 |
| 2 | 0.81 | (0.78, 0.83) | <0.0001 | 0.90 | (0.71, 1.13) | 0.3654 |
| 3 | 1.03 | (0.99, 1.07) | 0.1263 | 0.84 | (0.60, 1.19) | 0.3294 |
| ≥4 | 1.18 | (1.12, 1.25) | <0.0001 | 0.84 | (0.52, 1.36) | 0.4870 |
| **CCI** (ref =0) |  |  |  |  |  |  |
| ≥1 | 1.99 | (1.94, 2.04) | <0.0001 | 1.26 | (1.22, 1.29) | <0.0001 |
| **Coexisting comorbidities** |  |  |  |  |  |  |
| Hypertension | 2.63 | (2.57, 2.7) | <0.0001 | 1.09 | (1.06, 1.12) | <0.0001 |
| Hyperlipidemia | 1.00 | (0.96, 1.01) | 0.0650 | 1.19 | (1.07, 1.92) | <0.0001 |
| Coronary artery disease | 2.52 | (2.46, 2.58) | <0.0001 | 0.99 | (0.96, 1.02) | 0.3774 |
| Stroke | 3.95 | (3.85, 4.04) | <0.0001 | 1.49 | (1.45, 1.54) | <0.0001 |
| Depression | 2.06 | (1.98, 2.13) | <0.0001 | 1.51 | (1.45, 1.57) | <0.0001 |
| Anxiety | 1.80 | (1.75, 1.85) | <0.0001 | 1.17 | (1.13, 1.2) | <0.0001 |
| Heart failure | 3.17 | (3.06, 3.28) | <0.0001 | 1.03 | (0.99, 1.07) | 0.1888 |
| Peripheral vascular disease | 2.41 | (2.28, 2.56) | <0.0001 | 1.10 | (1.03, 1.16) | 0.0021 |
| COPD | 2.35 | (2.3, 2.41) | <0.0001 | 1.09 | (1.06, 1.12) | <0.0001 |
| Atrial fibrillation | 3.46 | (3.27, 3.66) | <0.0001 | 1.02 | (0.96, 1.08) | 0.6086 |
| Traumatic head injury | 1.87 | (1.8, 1.95) | <0.0001 | 1.46 | (1.4, 1.52) | <0.0001 |
| Hearing loss | 2.43 | (2.31, 2.55) | <0.0001 | 1.24 | (1.18, 1.31) | <0.0001 |
| Sleep apnea | 1.03 | (0.88, 1.2) | 0.6981 | 1.06 | (0.91, 1.24) | 0.4430 |
| Liver Cirrhosis | 1.14 | (1.11, 1.96) | <0.0001 | 1.11 | (1.04, 1.94) | <0.0001 |
| SLE | 1.66 | (1.54, 1.79) | <0.0001 | 1.10 | (1.02, 1.19) | 0.0114 |
| **Cigarette smoking** | 1.30 | (1.24, 1.36) | <0.0001 | 1.10 | (1.07, 1.24) | <0.0001 |
| **Alcohol liver diseases** | 1.02 | (1.00, 1.04) | 0.0722 | 1.19 | (1.06, 1.91) | <0.0001 |
| **Other Medications** |  |  |  |  |  |  |
| Statins | 0.82 | (0.81, 0.84) | <0.0001 | 0.88 | (0.83, 0.91) | <0.0001 |
| Anticholinergic drugs | 1.16 | (1.04, 1.88) | <0.0001 | 1.08 | (0.87, 1.10) | 0.7418 |
| Benzodiazepines | 1.09 | (0.67, 1.93) | 0.2050 | 1.14 | (0.77, 1.24) | 0.5243 |
| Antipsychotics | 1.01 | (0.95, 1.14) | 0.8098 | 1.00 | (0.89, 1.23) | 0.9679 |

**Abbreviations:** HR, hazard ratio; CI, confidence interval; CCI, Charlson comorbidity index; NTD, New Taiwan dollar; aDCSI, adapted diabetic complication severity index; COPD, Chronic Obstructive Pulmonary Disease; ref., reference group.

^*^Adjusted for all covariates shown in Table 1 using a Cox proportional regression model.

**Supplemental Figure 1.** Kaplan-Meier Curves for Cumulative Incidence of Dementia in Elderly Patients With Type 2 Diabetes Mellitus Stratified by Sarcopenia and Non-Sarcopenia after Propensity Score Matching

**
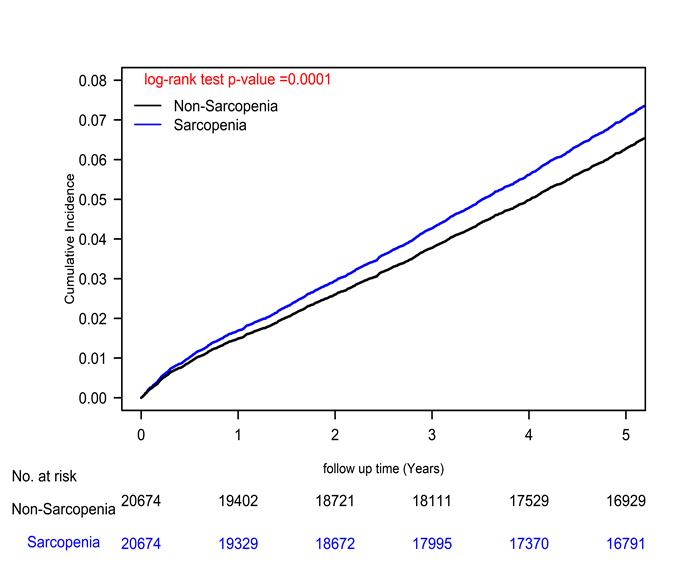
**
